# Metagenomic insights into isolable bacterial communities and antimicrobial resistance in airborne dust from pig farms

**DOI:** 10.3389/fvets.2024.1362011

**Published:** 2024-05-30

**Authors:** Si Thu Hein, Rangsiya Prathan, Songsak Srisanga, Dudsadee Muenhor, Thidathip Wongsurawat, Piroon Jenjaroenpun, Padet Tummaruk, Rungtip Chuanchuen

**Affiliations:** ^1^International Graduate Program of Veterinary Science and Technology, Faculty of Veterinary Science, Chulalongkorn University, Bangkok, Thailand; ^2^Research Unit in Microbial Food Safety and Antimicrobial Resistance, Department of Veterinary Public Health, Faculty of Veterinary Science, Chulalongkorn University, Bangkok, Thailand; ^3^Faculty of Environmental Management, Prince of Songkla University, Hat Yai, Songkhla, Thailand; ^4^Division of Medical Bioinformatics, Department of Research, Faculty of Medicine, Siriraj Hospital, Mahidol University, Bangkok, Thailand; ^5^Siriraj Long-Read Lab (Si-LoL), Faculty of Medicine, Siriraj Hospital, Bangkok, Thailand; ^6^Centre of Excellence in Swine Reproduction, Department of Obstetrics, Gynaecology, and Reproduction, Faculty of Veterinary Science, Chulalongkorn University, Bangkok, Thailand

**Keywords:** antimicrobial resistance, airborne dust, bacterial communities, metagenomic approach, pig farm

## Abstract

This study aims to investigate bacterial communities and antimicrobial resistance (AMR) in airborne dust from pig farms. Airborne dust, pig feces and feed were collected from nine pig farms in Thailand. Airborne dust samples were collected from upwind and downwind (25 meters from pig house), and inside (in the middle of the pig house) of the selected pig house. Pig feces and feed samples were individually collected from the pen floor and feed trough from the same pig house where airborne dust was collected. A direct total bacteria count on each sampling plate was conducted and averaged. The ESKAPE pathogens together with *Escherichia coli, Salmonella*, and *Streptococcus* were examined. A total of 163 bacterial isolates were collected and tested for MICs. Pooled bacteria from the inside airborne dust samples were analyzed using Metagenomic Sequencing. The highest bacterial concentration (1.9–11.2 × 10^3^ CFU/m^3^) was found inside pig houses. *Staphylococcus* (*n* = 37) and *Enterococcus* (*n* = 36) were most frequent bacterial species. *Salmonella* (*n* = 3) were exclusively isolated from feed and feces. Target bacteria showed a variety of resistance phenotypes, and the same bacterial species with the same resistance phenotype were found in airborne dust, feed and fecal from each farm. Metagenomic Sequencing analysis revealed 1,652 bacterial species across all pig farms, of which the predominant bacterial phylum was Bacillota. One hundred fifty-nine AMR genes of 12 different antibiotic classes were identified, with aminoglycoside resistance genes (24%) being the most prevalent. A total of 251 different plasmids were discovered, and the same plasmid was detected in multiple farms. In conclusion, the phenotypic and metagenomic results demonstrated that airborne dust from pig farms contained a diverse array of bacterial species and genes encoding resistance to a range of clinically important antimicrobial agents, indicating the significant role in the spread of AMR bacterial pathogens with potential hazards to human health. Policy measurements to address AMR in airborne dust from livestock farms are mandatory.

## 1 Introduction

Antimicrobial resistance (AMR) has been listed as one of the six global emerging environmental challenges by the United Nations Environment Programme (1). Transmission of AMR bacteria and their resistance determinants in environmental settings has been extensively researched in two primary habitats, aquaculture, and soils (2) e.g., lakes (3), soil with sewage and chicken manure (4), sediments, wastewater treatment plants (5), hospital wastewater (6). Prior studies have shown that ambient air contained a variety of bacteria and AMR determinants that may move long distances and across borders (7, 8). These place airborne AMR bacteria and their resistance determinants as an additional important route for the spread of AMR on a continental or global scale (8, 9), necessitating policies and regulation to reduce the spread of AMR through airborne dust.

WHO published a list of highly virulent and AMR bacterial pathogens as global targets for development of novel antibiotics, i.e., the ESKAPE pathogens (*Enterococcus faecium, Staphylococcus aureus, Klebsiella pneumoniae, Acinetobacter baumannii, Pseudomonas aeruginosa*, and *Enterobacter* spp.) that are also high priority organisms for AMR monitoring due to their ability to acquire high levels of resistance. The existence and spread of airborne AMR in ESKAPE pathogens in healthcare settings has been the subject of numerous research (10). The problem is far more extensive due to their expansion in animal farms and the environment (11). Transmission of AMR bacteria, including ESKAPE organisms, from animal farms to nearby communities through the air was demonstrated (12, 13).

The role of livestock farming, in particular pig farms, as a significant source and reservoir for AMR bacteria and determinants has been scientifically demonstrated (14). It is primarily caused by a high bacterial load and an increase in antimicrobial use at the farms, which creates a significant selective antimicrobial pressure and raises the risk of bacterial resistance development and spread. In addition to meat, soil and farm wastewater, airborne dust from pig farms has reportedly contained AMR bacteria and determinants, inevitably reaching the general population (7, 15). AMR bacteria-filled dust has the potential to become airborne and spread across the farms. It may also be released into the outside air by forced or natural ventilation, endangering neighboring people, farm animals, and the environment (16). To date, relatively few research has focused on AMR in ambient air, in comparison to food animals and their products. Despite the extensive antimicrobial use for a long time, little is still known about the bacterial community and AMR in airborne dust from livestock farms.

AMR studies typically rely on culture-based approaches, of which data on AMR phenotype and prevalence could be assessed. These approaches are usually time-consuming, laborious, and information limited. An advanced high-throughput technology and bioinformatic tool, Metagenomic sequencing methods, has proved the ways to overcome the limitations of the culture-based approach and to enhance the likelihood of finding AMR determinants, including novel resistance genes. It is anticipated that bacterial population and AMR traits in air samples from livestock farms would vary and be complex and therefore, culture-based methods and metagenomic sequencing analysis should be used in complementary for in-depth understanding.

The correlation between total levels of AMR and the use of antimicrobial agents was previously indicated (17). It was estimated that pigs consumed the highest antimicrobial quantity of 193 mg/PCU in 2017, accounting for 45% of the global increase in antimicrobial consumption from 2017 to 2030 (18). This is consistent with Thailand's 2020 One Health Report on Antimicrobial Consumption and AMR, which found that pigs consumed the highest amount of antibacterial agents in medicated feed (19). In addition, pig farm dust was previously shown to have higher levels of AMR bacteria and resistance genes in comparison to other livestock (20). Therefore, this study aimed to investigate bacterial communities and AMR in airborne pig farm dust.

## 2 Materials and methods

### 2.1 Sampling plan and location

This cross-sectional study was conducted in 9 pig farms located in Nakhon Sawan, Chainat, Supan Buri, Lopburi, and Saraburi of Thailand during the rainy season from late May to mid-October 2022 (Table 1). Samples were taken on a single visit to each farm. Due to the farm biosecurity, the selection of farms depended on the owners' willingness to participate in the study and the availability of veterinarians or animal health practitioners. All participating farms adopt a close house system with the number of pigs varying from 50 to 13,000. Three farms (Farm 4, Farm 6, and Farm 9) had large-scale commercial farming operations with between 11,000 and 13,000 pigs. Six farms were smaller scale farms, of which four farms had 2,000–5,000 pigs and another two farms had 50–200 pigs. Sampling was carried out at one pig house in each farm according to the owner of the farm's advice. For antimicrobial usage, amoxicillin was the most used antimicrobials by adding to feed either alone or in combination with tiamulin. Two farms (Farm 2 and Farm 3) declared using penicillin - streptomycin injection for treatment of ill pigs. Amoxicillin injection alone was used on one farm (Farm 2) for sick pigs. Information on the use of antimicrobial feed additives was unavailable.

**Table 1:** General details of the participating pig farms, average environmental parameters, bacterial concentrations, and number of bacterial species in airborne dust at different sampling positions in pig farms (*n* = 9).

**Farm No**.	**General information**			**Environmental parameters**					**Bacterial concentrations**			
	**Number of pigs**		**Stage of pig**	**Sample collection time**	**Wind speed (m/s)**	**Relative Humidity (%)/ Temperature (**°**C)**			**Total counts** ^a^			**No. of bacterial species** ^b^
	**Total in Farm**	**Sampling house**				**Upwind**	**Inside**	**Downwind**	**Upwind**	**Inside**	**Downwind**	**Inside**
1	5,000	120	Sow, piglets	May	2.9	87.3/28.9	94.2/27.7	93.0/28.0	0.7	5.0	0.2	ND
2	5,000	120	Sow, piglets	July	0.5	92.1/27.8	94.4/27.6	89.8/28.2	2.2	3.8	3.7	ND
3	3,000	140	Sow	September	0.8	75.7/30.8	74.2/30.6	84.7/28.3	1.4	2.4	0.6	1293
4	13,000	700	Fattener	September	1.1	79.2/30.9	80.3/30.4	82.2/30.2	0.8	11.2	1.3	476
5	200	200	Fattener^†^	September	1.4	72.8/31.0	68.9/31.6	73.2/30.1	0.9	6.9	3.1	325
6	11,000	320	Fattener	October	1.7	63.8/31.9	80.3/28.4	63.8/31.4	1.6	7.3	3.9	256
7	42	42	Boar	October	1.9	57.5/34.4	63.0/31.5	63.2/32.0	0.2	2.5	1.7	499
8	2,000	250	Fattener	October	2.2	40.2/38.2	62.1/29.8	51.4/35.5	0.3	1.9	2.1	669
9	11,000	400	Fattener	October	2.5	52.9/35.1	48.4/34.7	40.7/34.7	0.5	1.9	3.7	341
Average		-	-	-	-	-	-	-	1.0	4.8	2.3	-

^†^Open-housed system. ^a^Determined by direct plate counts ( × 10^3^ CFU/m^3^ of air volume). ^b^Based on metagenomic analysis of inside farm samples.

### 2.2 Airborne dust sample collection

Airborne dust samples were collected using a BioStage^®^ single-stage viable cascade impactor (SKC Inc., Eighty-Four, PA, USA) with 400 pores of 0.6 μm in size and a Quick Take 30 pump with a flow rate of 28.3 L/min. Tryptic soy agar (TSA, Difco^TM^, MD, USA) in 100 mm × 15 mm petri dishes were placed inside the impactor for collecting dust. The impactor was positioned approximately 1.5 m above the ground, which is a person's average respiratory height (21). Airborne dust samples were taken from the selected pig house at three separate locations, inside (at the center of the pig house), upwind (25 m from the pig house), and downwind (25 m from the pig house). Sampling time was 2 min per plate. Three TSA plates were utilized to collect airborne dust samples at each location for a total of nine plates per farm.

The Kestrel 3000 Weather Meter (Nielsen-Kellermen, PA, USA) was used to record the environmental variables potentially affecting the composition and diversity of bacterial communities, including average wind speed (m/s), ambient air temperature (°C), and relative humidity (%) at each sampling site (Table 1).

### 2.3 Collection of pig feces and feed samples

Pig feces and feed samples at least 25 g of each were taken from the same pig house where airborne dust was collected. Fecal droppings were collected from the pen floor, and feed was collected from feeding trough. The samples were immediately placed in an ice box and transported to the laboratory within 6 h of collection.

### 2.4 Quantification of total bacteria

Total bacteria were directly counted on TSA plates after a 24-h incubation at 37°C (22). The average colony-forming units per cubic meter of air (CFU/m^3^) were calculated from the counting results of three plates at each sampling position. Bioaerosol concentrations were estimated using the following formula (23).

Bioaerosol concentration (CFU/m^3^) = Number of colonies / (Adjusted flow rate of the sampling machine × Sampling duration in minutes).

### 2.5 Isolation and identification of target bacteria

The pooled bacteria from all three TSA plates obtained from each sampling position, pig feed samples (25 g each) and feces samples (25 g each) were separately pre-enriched in Buffer Peptone Water (BPW) and incubated at 37°C for 24 h. Then, target bacterial species were isolated and identified using previously published protocols, including the ESKAPE bacteria (i.e., *Enterococcus* species (24, 25), *Staphylococcus* species (26), *Klebsiella* species (27, 28), *Acinetobacter* (29), *Pseudomonas* species (30), *Enterobacter* species (27, 31), *Escherichia coli* (24, 32), *Salmonella* (33), and *Streptococcus* species (34, 35). One loopful of bacteria pooled from the three agar plates was steaked on selective media, *Klebsiella* species, *Enterobacter* species and *Escherichia coli*, MacConkey agar (Difco^TM^ & BBL^TM^); *Enterococcus* species, Slanetz & Bartley agar (OXOID^®^, Hampshire, UK); *Staphylococcus* species, mannitol salt agar (BBL^TM^, MD, USA); *Acinetobacter* species, CHROMagar™ Acinetobacter (CHROMagar, Paris, France); *Pseudomonas* species, *Pseudomonas* Agar Base (Difco^TM^, MI, USA); *Salmonella*, Xylose Lysine Deoxycholate (XLD) agar (Difco^TM^) and *Streptococcus* species, Columbia agar supplemented with 5% sheep blood agar (Difco^TM^) and incubated at 37°C for 18–24 h.

Typical colonies of *E. coli* from MacConkey agar were streaked on Eosin Methylene Blue (EMB) agar (Difco^TM^) and confirmed by Indole test. Typical *Salmonella* colonies from XLD plates were confirmed by growth in Triple sugar iron agar (TSI) (Difco^TM^) and Motility Indole Lysine (MIL) agar (Difco^TM^). *Enterococcus* species, *Klebsiella* species, *Enterobacter* species, and *Streptococcus* species were confirmed by PCR using the following primer sets; *Enterococcus* species, EN-1 5′-TACTGACAAACCATTCATGATG-3′ and EN-2 5′-AACTTCGTCACCAACGCGAAC-3′; *Klebsiella* species, KL-1 5′-CGCGTACTATACGCCATGAACGTA-3′ and KL-2 5′-ACCGTTGATCACTTCGGTCAGG-3′; *Enterobacter* species, Ent-1 5′-GGCAAAGCTCAACCCGGAGGTATTCT-3′ and Ent-2 5′-CAAAGAAAGATAATAATTTCACGGTTAGTC-3′ and *Streptococcus* species, C-1 5′-GCGTGCCTAATACATGCAA-3′ and C-2 5′-TACAACGCAGGTCCATCT-3′.

A single purified colony of each bacterial species was collected from each sampling position in each pig house and stored as a 20% glycerol stock at −80°C for further analysis.

### 2.6 Antimicrobial susceptibility testing

A total of 163 bacterial isolates (*n* = 163) were collected (Table 4) and tested for their antimicrobial susceptibilities by broth microdilution method using the Sensititre^TM^ Complete Automated AST System (Thermo Fisher Scientific, MA, USA). Different Sensititre^TM^ MIC plates were used for different bacteria as follows: EUVSEC2, ASSECAF, and ASSECB for *E. coli, Salmonella, Klebsiella* species, and *Enterobacter* species; GNX2F for *Acinetobacter* and *Pseudomonas* species; EUST2 for *Staphylococcus* species and STP67 for *Streptococcus* species. All antibiotic plates were purchased from Trek Diagnostic Systems, West Sussex, UK. Two-fold agar dilution method was used for the susceptibility testing of *Enterococcus* species (36). The CLSI interpretive criteria was used for defining bacterial isolates as resistant or susceptible (36). *E. coli* ATCC 25922, *P. aeruginosa* ATCC 27853, *S. aureus* ATCC 29213 and *E. faecalis* ATCC 29212 served as quality control.

### 2.7 Metagenomic sequencing analysis

A loopful of the pooled airborne dust samples was taken directly from the pooled three TSA plates collected at the inside position of each pig house (*n* = 7) before the enrichment for isolation of target bacterial species to extract genomic DNA using ZymoBIOMICS^TM^ DNA Miniprep Kit (Zymo Research, Irvine, CA, USA) following the manufacturer's instructions. The genomic DNA from Farm 1 and 2 samples were of poor quality and unusable for Metagenomic Sequencing. The concentration and quality of the extracted DNA were measured using a NanoDrop ND 1000 spectrophotometer (Thermo Fisher Scientific). The quality and degradation were additionally evaluated by running 1 μl of the genomic DNA on 0.8% agarose gel electrophoresis stained with RedSafe nucleic acid staining solution (Thermo Fisher Scientific). The genomic DNA was submitted for Metagenomic Sequencing at Siriraj Long-read Lab (Si-LoL), Faculty of Medicine Siriraj Hospital, Mahidol University, Bangkok, Thailand, using an Oxford Nanopore sequencing platform. The Metagenomic Sequencing results were analyzed as previously described (37). Briefly, the output fastq files were uploaded to BugSeq workflow v20.07.1 (https://bugseq.com) for metagenomic classification. The reads were quality controlled with fastp v20.07.1, a FASTQ data pre-processing tool, using a minimum average read quality of Phred 7, a minimum read length of 100 bp, and the default low complexity filter. The reads were then mapped with minimap2 v2.17 to identify consensus with all microbes in the NCBI nt database. The obtained results were visualized in MultiQC reporting tool. AMR genes were identified by aligning the reads against the Resfinder (http://genomicepidemiology.org) with minimap2. Analysis from BugSeq outputs and visualizations were performed in Pavian R package v1.2.0 in RStudio (R version 4.1.0) (https://www.r-project.org/). Kraken 2 was used to analyze bacterial species and visualize the Pavian R package results.

## 3 Results

### 3.1 Abundance of airborne bacteria

The highest average total bacterial counts were observed inside the pig houses (4.8 × 10^3^ CFU/m^3^, 1.9–11.9 × 10^3^ CFU/m^3^) (Table 1), followed by the downwind positions (2.3 × 10^3^ CFU/m^3^, 0.2–3.9 × 10^3^ CFU/m^3^) and the upwind positions (1.0 × 10^3^ CFU/m^3^, 0.3–2.2 × 10^3^ CFU/m^3^). The inside pig houses had higher bacterial loads in most farms, except for Farms 8 and 9, where the downwind concentrations were higher. The bacterial concentration inside the pig house at Farm 4 was highest (11.2 × 10^3^ CFU/m^3^), while that of Farm 7 was lowest (2.5 × 10^3^ CFU/m^3^). Only Farm 2 had comparable concentrations at the inside and downwind.

### 3.2 Bacterial species isolated from airborne dust in pig farms

Using conventional-standard methods, the abundance and distribution of the ESKAPE species, *E. coli, Salmonella* and *Streptococcus* species were determined (Table 2). *Staphylococcus* (*n* = 37) and *Enterococcus* species (*n* = 36) were the most frequently detected bacterial species in all samples. Of all five *Klebsiella* isolates, two were obtained from airborne samples, while the others were isolated from feed samples. *Salmonella* species (*n* = 3) were found only in feed and feces but not in the airborne dust samples. Only one *Pseudomonas* isolate, which came from the feed sample, was obtained.

**Table 2:** Bacterial species identified at different sampling positions in pig farms (*n* = 175).

**Farms**	**Bacterial Species** ^**a, b**^									**Total**
	***Enterococcus***	***Staphylococcus***	***Klebsiella***	***Acinetobacter***	***Pseudomonas***	***Enterobacter***	***E. coli***	***Salmonella***	***Streptococcus***	
Farm 1	U, I, D, Fe, Fa	U, I, D, Fe	N	Fa	N	N	U, I, D, Fa	N	N	14
Farm 2	U, I, D, Fe, Fa	U, I, D, Fe, Fa	Fe	D	N	N	I, D, Fa	N	U, I, D, Fe, Fa	21
Farm 3	U, I, D, Fa	U, I, D, Fa	Fe	U, I, D, Fe, Fa	N	N	U, Fe	N	U, I, D, Fe, Fa	20
Farm 4	U, Fa	U, I, D, Fe, Fa	U	I, D, Fe, Fa	N	N	U, I, Fe, Fa	N	N	16
Farm 5	Fe, Fa	U, I, D, Fe	I	U, D, Fe, Fa	Fe	N	Fe, Fa	N	N	14
Farm 6	I, D, Fe, Fa	I, D, Fe	N	U, I, D, Fe, Fa	N	I, Fa	I	N	N	15
Farm 7	U, I, D, Fe, Fa	U, I, D, Fe, Fa	N	I, D, Fa	N	I, Fe, Fa	I, Fe, Fa	Fa	I, D, Fe, Fa	24
Farm 8	U, I, D, Fe, Fa	I, Fe	N	U, I, D, Fe	N	I, Fe, Fa	I, Fe	Fe	N	17
Farm 9	U, I, D, Fa	U, I, D, Fe, Fa	Fe	U, I, D, Fe, Fa	N	Fe	Fe, Fa	Fa	I, Fe, Fa	22
Total	36	37	5	32	1	9	23	3	17	163

^a^U, Upwind; I, Inside; D, Downwind; Fe, Feed; Fa, Feces; N, Not found. ^b^One isolate was collected for each bacterial species from every sampling position within each pig house.

### 3.3 Phenotypic antimicrobial susceptibilities

Overall, the bacterial isolates obtained from pig farm environment in this study exhibited various AMR phenotype and rates (Table 3). The number of target bacterial species varied greatly amongst the farms, therefore the comparison between farms is not appropriate.

**Table 3:** Antimicrobial resistance rates (%) of bacterial species isolated from airborne dust, feed, and feces from the pig farms.

**Antimicrobials**	**No. of resistant isolates (%)**								
	***Enterococcus*** **(*****n** =* **36)**	***Staphylococcus*** **(*****n** =* **37)**	***Klebsiella*** **(*****n** =* **5)**	***Acinetobacter*** **(*****n** =* **32)**	***Pseudomonas*** **(*****n** =* **1)**	***Enterobacter*** **(*****n** =* **9)**	***E. coli*** **(*****n** =* **23)**	***Salmonella*** **(*****n** =* **3)**	***Streptococcus (n** = **17)***
Amoxicillin/Clavulanic Acid	-	-	-	-	-	-	-		3 (18)
Ampicillin	2 (6)	-	5 (100)	-	-	9 (100)	23 (100)	3 (100)	-
Azithromycin	-	-	5 (100)	-	-	9 (100)	-	2 (67)	13 (76)
Aztreonam	-	-	-	0 (0)	1 (100)	-	-		-
Cefepime	-	-	2 (40)	2 (6)	0 (0)	2 (22)	3 (13)	1 (33)	8 (47)
Cefotaxime	-	-	3 (60)	14 (42)	1 (100)	4 (44)	9 (39)	2 (67)	6 (35)
Cefoxitin	-	14 (38)	3 (60)	-	-	9 (100)	3 (13)	1 (33)	-
Ceftazidime	-	-	2 (40)	8 (24)	0 (0)	2 (22)	9 (39)	1 (33)	-
Cefuroxime (sodium)	-	-	-	-	-	-	-		10 (59)
Chloramphenicol	12 (33)	12 (32)	3 (60)	-	-	8 (89)	17 (74)	2 (67)	6 (35)
Ciprofloxacin	-	14 (38)	1 (20)	12 (36)	1 (100)	2 (22)	3 (13)	0 (0)	-
Clindamycin	-	32 (86)	-	-	-	-	-		-
Colistin	-	-	1 (20)	8 (24)	0 (0)	7 (78)	4 (17)	1 (33)	-
Doripenem	-	-	-	2 (6)	0 (0)	-	-		-
Doxycycline	-	-	-	16 (48)	1 (100)	-	-		-
Ertapenem	-	-	0 (0)	-	-	0 (0)	1 (4)	0 (0)	3 (18)
Erythromycin	34 (94)	28 (76)	-	-	-	-	-		12 (71)
Fusidate	-	17 (46)	-	-	-	-	-		-
Gentamicin	7 (19)	13 (35)	3 (60)	17 (52)	1 (100)	6 (67)	12 (52)	1 (33)	-
Impipenem	-	-	-	2 (6)	0 (0)	-	-		-
Kanamycin	-	9 (24)	-	-	-	-	-		-
Levofloxacin	-	-	-	6 (18)	0 (0)	-	-		0 (0)
Linezolid	-	6 (16)	-	2 (6)	0 (0)	-	-		-
Nalidixic Acid			0 (0)			1 (11)	3 (13)	0 (0)	-
Meropenem	-	-	-	-	-	-	-		2 (12)
Minocycline	-	-	-	13 (39)	1 (100)	-	-		-
Moxifloxacin	-	-	-	-	-	-	-		2 (12)
Mupirocin	-	0 (0)	-	-	-	-	-		-
Penicillin	-	35 (95)	-	-	-	-	-		15 (88)
Polymyxin B	-	-	-	8 (24)	0 (0)	-	-		-
Quinupristin/dalfopristin	-	29 (78)	-	-	-	-	-	-	-
Rifampin	-	0 (0)	-	-	-	-	-	-	-
Streptomycin	17 (47)	29 (78)	-	-	-	-	-	-	-
Sulfamethoxazole	-	4 (11)	0 (0)	-	-	0 (0)	18 (78)	0 (0)	-
Temocillin			0 (0)			0 (0)	4 (17)	1 (33)	
Tetracycline	33 (92)	30 (81)	5 (100)	-	-	7 (78)	17 (74)	3 (100)	12 (71)
Tiamulin	-	37 (100)	-	-	-	-	-	-	-
Ticarcillin/ Clavulanic Acid	-	-	-	5 (15)	1 (100)	-	-	-	-
Tigecycline			0 (0)			0 (0)	17 (74)	3 (100)	
Tobramycin	-	-	-	8 (24)	0 (0)	-		-	-
Trimethoprim	-	24 (65)	2 (40)	-	-	7 (78)	16 (70)	3 (100)	-
Trimethoprim/ Sulfamethoxazole	-	-	-	23 (70)	1 (100)	-	-	-	8 (47)
Vancomycin	0 (0)	3 (8)	-	-	-	-	-	-	0 (0)

(-) Not detected.

High resistance rates to erythromycin (94%) and tetracycline (92%) were observed for the *Enterococcus* isolates from airborne dust, feed, and feces (*n* = 36). The *Staphylococcus* isolates (*n* = 37) showed high resistance rates to tiamulin (100%), penicillin (95%), and clindamycin (86%). Notably, *Staphylococcus* isolates collected from airborne dust displayed resistance to a wider range of antibiotics compared to those from feed and feces samples.

All the *E. coli* isolates (*n* = 23) were resistant to ampicillin, while displayed high resistance rates to most antimicrobials tested. All three *Salmonella* isolates were resistant to ampicillin, tetracycline, tigecycline and trimethoprim. They also exhibited resistance to cefotaxime, cefoxitin but none were resistant to ciprofloxacin. The *Streptococcus* isolates (*n* = 17) showed high resistance rates to penicillin (88%), azithromycin (76%), erythromycin (71%), and tetracycline (71%). All *Klebsiella* species (*n* = 5) obtained from airborne dust and feed samples were resistant to ampicillin, azithromycin, and tetracycline, while some were resistant to trimethoprim/sulfamethoxazole (16/5, 70%) and gentamicin (12/5, 52%).

The only one isolate of *Pseudomonas* species, obtained from a feed sample of Farm 5, was resistant to a wide range of antimicrobials, including aztreonam, cefotaxime, and ciprofloxacin. *Enterobacter* isolates (*n* = 9) demonstrated resistance to most antimicrobials, including ampicillin, azithromycin, cefoxitin, chloramphenicol, and gentamicin, with high resistance rates ranging from 89% to 100%. Resistance to cefepime, cefotaxime, and ceftazidime was also observed at a lower frequency.

The bacteria isolate with various AMR phenotypes widely distributed among farms (Table 4). For each farm, the same bacterial species with the same resistance phenotype were found in airborne dust, feed, and fecal samples, for example, *Enterococcus* resistant to ampicillin, azithromycin, cefotaxime, ceftazidime, chloramphenicol, gentamicin, tigecycline and trimethoprim were found in airborne dust, feed and fecal samples obtained from Farm 3.

**Table 4:** Phenotypic characteristics of antimicrobial resistance in target bacterial species isolated from airborne dust, feed, and feces in pig farms.

**Bacterial species**	**Sampling sources (*n =* )**	**Farms**								
		**Farm 1**	**Farm 2**	**Farm 3**	**Farm 4**	**Farm 5**	**Farm 6**	**Farm 7**	**Farm 8**	**Farm 9**
***Enterococcus*** **(*****n** **=*** **36)**	**Airborne (22)**	ERY, TET	ERY, TET, STR	CHL, ERY, TET, GEN, STR	CHL, ERY, TET, GEN, STR	NA	AMP, ERY, TET, GEN, STR	ERY, TET, STR	ERY, TET	CHL, ERY, TET, GEN, STR
	**Feed (6)**	ERY, TET	ERY, TET, STR	NA	NA	CHL, TET, GEN	ERY, TET	ERY	ERY, TET	NA
	**Feces (8)**	CHL, ERY, TET, STR	CHL, ERY, TET	AMP, CHL, ERY, TET, STR	CHL, ERY, TET, GEN, STR	S	ERY, TET	ERY, TET	CHL, ERY, TET, STR	CHL, ERY, TET, STR
***Staphylococcus*** **(*****n** **=*** **37)**	**Airborne (24)**	CIP, CLI, ERY, FUS, GEN, KAN, PEN, STR, TET, TIA, TRI	FOX, CIP, CLI, ERY, FUS, PEN, SYN, STR, TET, TIA, TRI	CHL, CIP, CLI, ERY, FUS, GEN, KAN, LZD, PEN, SYN, STR, TET, TIA, TRI	FOX, CHL, CIP, CLI, ERY, FUS, GEN, KAN, PEN, SYN, STR, TET, TIA, TRI	FOX, CHL, CIP, CLI, ERY, FUS, GEN, PEN, SYN, STR, TET, TIA, TRI	CIP, CLI, ERY, GEN, PEN, SYN, STR, TET, TIA, TRI	CLI, ERY, FUS, PEN, SYN, STR, TET, TIA, TRI	FOX, CIP, CLI, ERY, GEN, PEN, SYN, STR, TET, TIA, TRI	FOX, CHL, CIP, CLI, ERY, FUS, GEN, PEN, SYN, STR, TET, TIA, TRI
	**Feed (8)**	CLI, ERY, FUS, PEN, SYN, TIA, TRI	FOX, CHL, CLI, ERY, PEN, SYN, TIA	NA	FOX, CHL, CIP, CLI, ERY, FUS, GEN, KAN, LZD, PEN, SYN, SMX, TET, TIA, TRI, VAN	FOX, CHL, CLI, ERY, FUS, GEN, LZD, PEN, SYN, STR, SMX, TET, TIA, TRI	FOX, CLI, ERY, PEN, SYN, STR, TET, TIA	CLI, ERY, PEN, SYN, STR, TET, TIA, TRI	FOX, CLI, ERY, PEN, SYN, TET, TIA	FOX, CLI, ERY, PEN, SYN, STR, SMX, TET, TIA, TRI
	**Feces (5)**	NA	CLI, ERY, FUS, PEN, SYN, SYN, STR, TIA, TRI	CLI, ERY, FUS, KAN, PEN, SYN, STR, TET, TIA	CHL, CLI, ERY, GEN, KAN, LZD, PEN, SYN, STR, TET, TIA, TRI	NA	NA	FOX, CIP, CLI, ERY, FUS, PEN, SYN, STR, TET, TIA, TRI		FOX, CHL, CLI, ERY, FUS, GEN, LZD, PEN, SYN, STR, SMX, TET, TIA, TRI, VAN
***Klebsiella*** **(*****n** **=*** **5)**	**Airborne (2)**	NA	NA	NA	AMP, AZI, FEP, CTX, CAZ, CHL, GEN, TET	AMP, AZI, FEP, CTX, FOX, CAZ, CHL, GEN, TET	NA	NA	NA	NA
	**Feed (3)**	NA	AMP, AZI. FOX, CHL, TET, TRI	AMP, AZI	NA	NA	NA	NA	NA	AMP, AZI, CTX, FOX, CIP, CST, GEN, TET, TRI
***Acinetobacter*** **(*****n** **=*** **32)**	**Airborne (19)**	NA	DOX, GEN, MIN, TOB, SXT	FEP, CTX, CAZ, CIP, CST, DOR, DOX, GEN, IMI, LVX, MER, MIN, POL, TOB, SXT	CTX, DOX, GEN, MIN, SXT	S	CTX, CAZ, CIP, CST, DOX, GEN, MIN, POL, TCC, SXT	CIP, SXT	FEP, CTX, CAZ, CIP, CST, DOX, GEN, MIN, POL, TCC, TOB, SXT	CTX, CIP, CST, DOR, DOX, IMI, MER, POL, TOB, SXT
	**Feed (6)**	NA	NA	CIP, DOX, GEN, LVX, SXT	CTX, CAZ, CIP, CST, DOX, GEN, LVX, MIN, POL, TCC, TOB, SXT	CAZ, DOX, GEN, SXT	S	NA	CTX, CAZ, TCC	CTX, CST, DOX, GEN, MIN, POL, TCC, SXT
	**Feces (7)**	CTX, MIN	NA	CTX, DOX, GEN, MIN, SXT	CAZ, CIP, DOX, GEN, LVX, MIN, TOB, SXT	CAZ, DOX, GEN, SXT, TOB, SXT	CTX, CIP, CST, DOX, GEN, LVX, MIN, POL, TOB, SXT	CTX, CIP	DOX, GEN, MIN, SXT	CTX, CIP, DOX, GEN, LVX, MIN, SXT
***Pseudomonas*** **(*****n** **=*** **1)**	**Feed**	NA	NA	NA	NA	AZT, CTX, CIP, DOX, GEN, MIN, TCC, SXT	NA	NA	NA	NA
***Enterobacter*** **(*****n** **=*** **9)**	**Airborne (3)**	NA	NA	NA	NA	NA	AMP, AZI, FOX, CHL, CST, TET	AMP, AZI, FEP, CTX, FOX, CAZ, CHL, CST, GEN, TET, TRI	AMP, AZI, FEP, CTX, FOX, CAZ, CHL, CST, GEN, TET, TRI	NA
	**Feed (2)**	NA	NA	NA	NA	NA	AMP, AZI, CTX, FOX, CHL, CST, GEN, TET, TRI	AMP, AZI, FOX, TET	AMP, AZI, FOX, CHL, CIP, CST, GEN, NAL, TET, TRI	AMP, AZI, FOX, CHL, CST, GEN, TRI
	**Feces (4)**	NA	NA	NA	NA	NA	NA	AMP, AZI, FOX, CHL, TRI	AMP, AZI, CTX, FOX, CHL, CIP, CST, GEN, TET, TRI	NA
***E. coli*** **(*****n** **=*** **23)**	**Airborne (10)**	AMP, CTX, FOX, CAZ, ETP, SMX, TEM, TET, TGC, TRI	AMP, TGC	AMP, FEP, CTX, CAZ, CHL, GEN, SMX, TET, TGC, TRI	AMP, FEP, CTX, CAZ, CHL, GEN, SMX, TET, TGC, TRI	NA	AMP, CHL, CIP, CST, NAL, SMX, TET, TRI	AMP, CTX, CAZ, CHL, CST, GEN, SMX, TET, TGC, TRI	AMP, CHL, CST, GEN, SMX, TET, TGC, TRI	NA
	**Feed (6)**	NA	NA	AMP, FOX, CHL, SMX, TEM, TET, TGC	AMP, CTX, CAZ, CHL, GEN, TGC, TRI	AMP, FOX, CHL, GEN, SMX, TET, TGC, TRI	NA	AMP, CHL, SMX, TET, TGC	AMP, CHL, GEN, SMX, TET, TGC, TRI	AMP, CHL, CIP, CST, GEN, SMX, TET, TRI
	**Feces (7)**	AMP, CHL, SMX, TEM, TET, TGC, TRI	AMP, CHL, SMX, TET, TGC, TRI	NA	AMP, CTX, CAZ, CHL, GEN, NAL, SMX, TET, TGC, TRI	AMP, FEP, CTX, CAZ, CHL, CIP, GEN, NAL, SMX, TEM, TET, TGC, TRI	NA	AMP, CHL	NA	AMP, GEN, SMX, TET, TRI
***Salmonella*** **(*****n** **=*** **3)**	**Feed (1)**	NA	NA	NA	NA	NA	NA	NA	AMP, FEP, CTX, FOX, CAZ, TEM, TET, TGC, TRI	NA
	**Feces (2)**	NA	NA	NA	NA	NA	NA	AMP, AZI, CHL, TET, TGC, TRI	NA	AMP, AZI, CTX, CHL, CST, GEN, TET, TGC, TRI
***Streptococcus** (*n =* 17)*	**Airborne (9)**	NA	AZI, FEP, CTX, FUR, ETP, ERY, PEN TET, SXT	AZI, FEP, CTX, FUR, ETP, ERY, MEM, PEN, TET, SXT	NA	NA	NA	AZI, FEP, PEN	NA	AZI, FEP, CHL, ERY, PEN
	**Feed (4)**	NA	AML, AZI, FEP, CTX, FUR, CHL, ERY, PEN, SXT	FUR, ERY, PEN, TET	NA	NA	NA	AZI, FEP, CTX, FUR, ETP, ERY, MEM, PEN, TET	NA	AML, AZI, FUR, CHL, ERY, MXF, PEN, TET, SXT
	**Feces (4)**	NA	AZI, FEP, FUR, CHL, ERY, PEN, TET	AZI, FEP, CTX, FUR, CHL, ERY, PEN, TET, SXT	NA	NA	NA	AZI, ERY, PEN, TET, SXT	NA	AML, AZI, CTX, FUR, CHL, ERY, MXF, PEN, TET, SXT

One isolate was collected from each positive sample. AML, Amoxicillin/Clavulanic Acid; AMP, Ampicillin; AZI, Azithromycin; AZT, Aztreonam; CAZ, Ceftazidime; CHL, Chloramphenicol; CIP, Ciprofloxacin; CLI, Clindamycin; CST, Colistin; CTX, Cefotaxime; DOR, Doripenem; DOX, Doxycycline; ERY, Erythromycin; ETP, Ertapenem; FEP, Cefepime; FOX, Cefoxitin; FUR, Cefuroxime (sodium); FUS, Fusidate; GEN, Gentamicin; IMI, Impipenem; KAN, Kanamycin; LVX, Levofloxacin; LZD, Linezolid; MER, Meropenem; MIN, Minocycline; MXF, Moxifloxacin; NAL, Nalidixic Acid; PEN, Penicillin; POL, Polymyxin B; SMX, Sulfamethoxazole; STR, Streptomycin; SXT, Trimethoprim/ Sulfamethoxazole; SYN, Quinupristin/dalfopristin; TCC, Ticarcillin/ Clavulanic Acid; TEM, Temocillin; TET, Tetracycline; TGC, Tigecycline; TIA, Tiamulin; TMP, Trimethoprim; TOB, Tobramycin; VAN, Vancomycin; NA, Not found: S, Susceptible.

### 3.4 Metagenomic characteristics of airborne dust inside pig farms

#### 3.4.1 Bacterial community compositions

**Figure 1:**
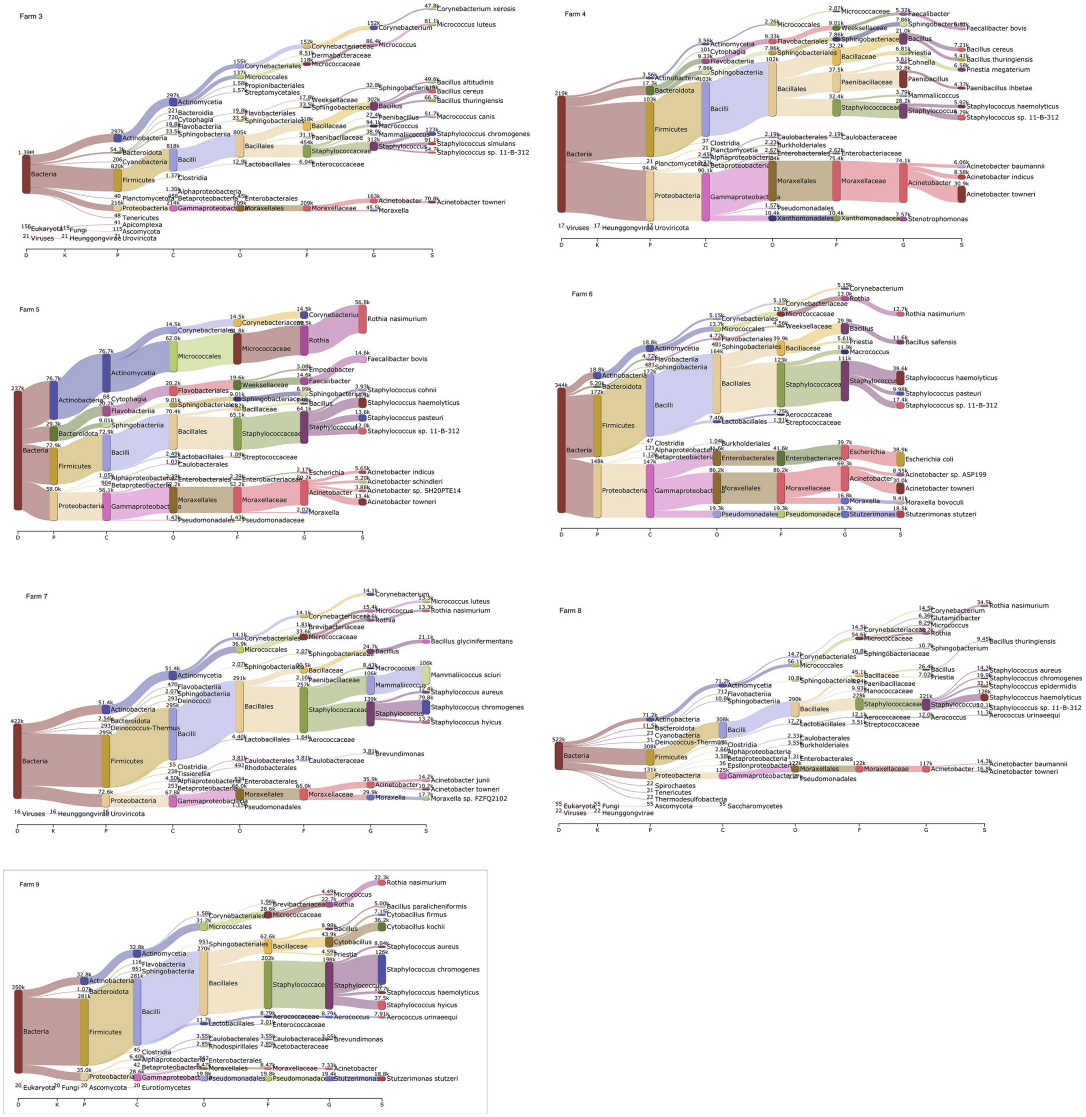
Sankey visualization from the Kraken 2 analysis of airborne bacterial communities inside pig farms (*n* = 7). It is shown in different taxonomy levels. The higher the portion for each phylum, the higher read counts. The number indicates the read counts. D, Domain; K, Kingdom; P, Phylum; C, Class; O, Order; F, Family; G, Genus; S, Species.

**Figure 2:**
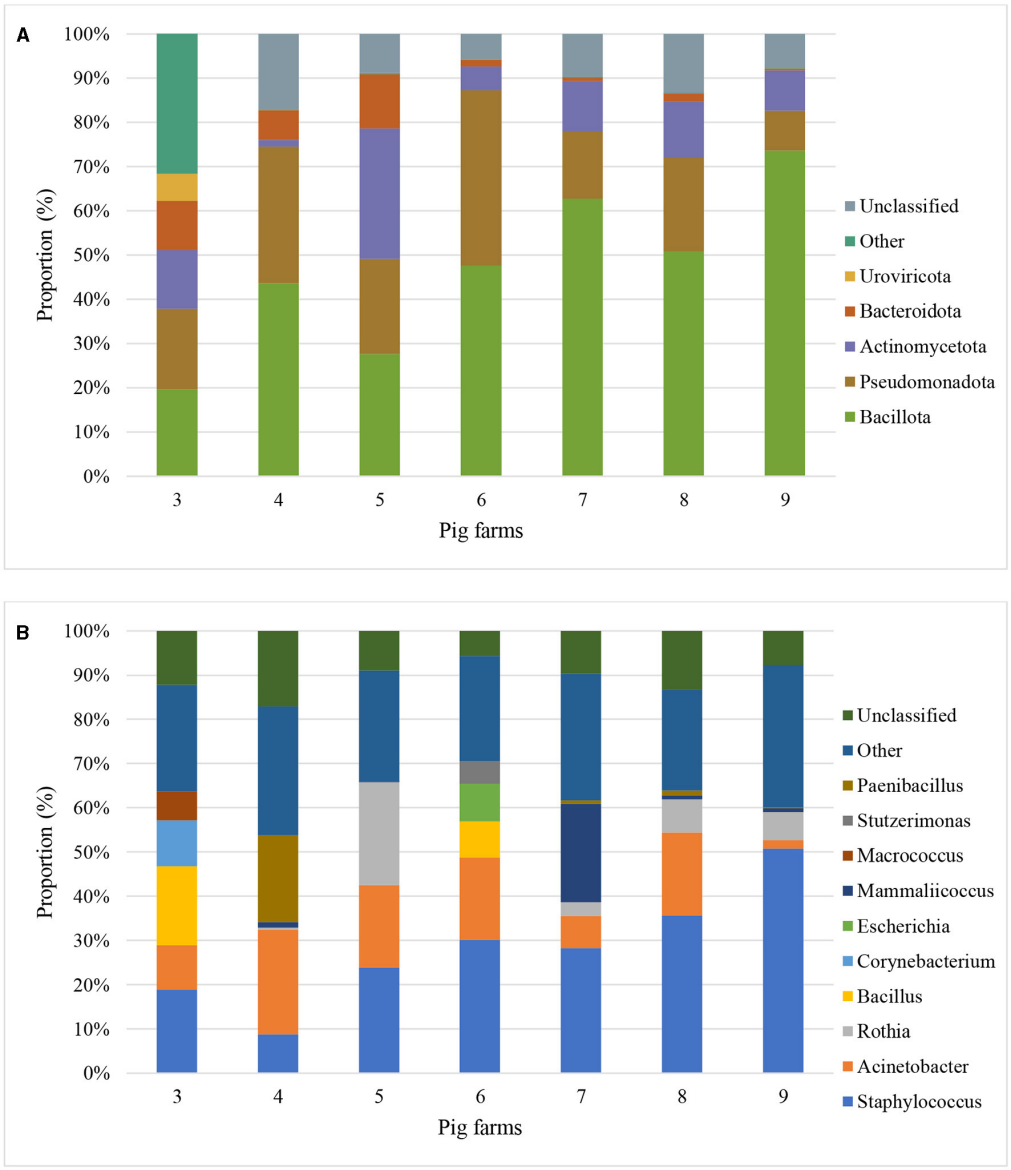
Metagenomic classifications of bacterial community compositions at Phylum level (**A)** and Genus level (**B)** of airborne dust inside seven pig farms by Proportion (percentage of sequencing reads that align or map to a specific phyla and genus to the total number of reads) of top 5 phyla and genus.

Based on the Kraken 2 analysis, a total number of 1,652 bacterial species were found in all seven pig farms (Supplementary Table S1), of which *Staphylococcus chromogenes* was the most common bacterial species, followed by *Mammaliicoccus sciuri* and *Staphylococcus haemolyticus*. The Sankey visualization revealed that the predominant bacterial phyla observed inside pig house were Bacillota and Pseudomonadota (Figure 1). The Burkholderales order was discovered in Farms 4, 6, and 8. The relative proportions of phyla and genus of bacterial community compositions present in airborne dust from all pig farms are shown in Figure 2. The Bacillota phylum occupied the highest proportion of all farms (19.7–73.7%), of which Farm 9 had the highest percentage (73.7%). *Staphylococcus* was the most detected genus (50.8%). This bacterial pathogen was identified in all farms, except for Farm 4 where *Acinetobacter* was predominant. The number of bacterial species identified in each farm varied from 1,293 in Farm 3 to 325 in Farm 5 (Table 1).

#### 3.4.2 Antimicrobial resistance genes

**Figure 3:**
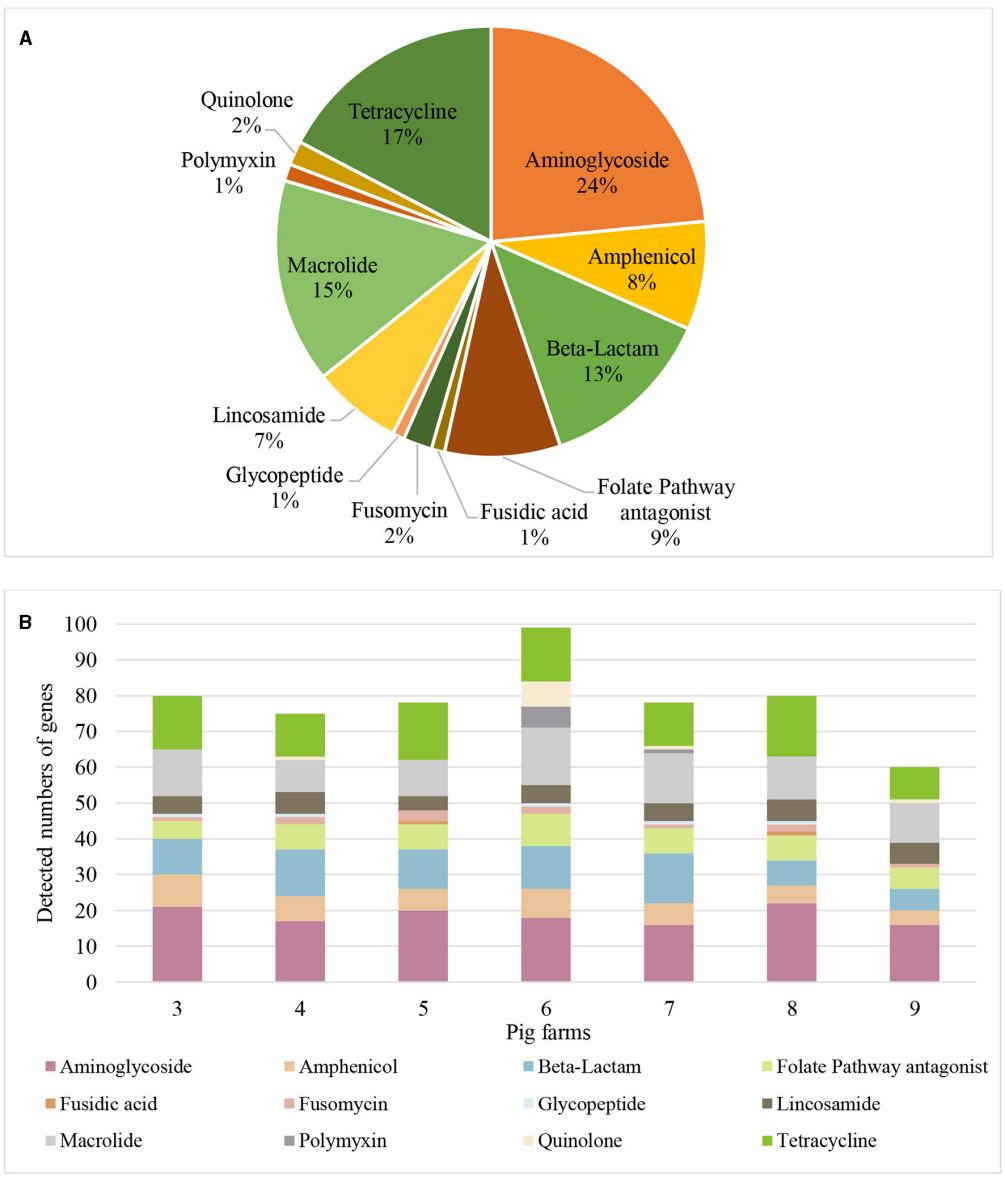
Antimicrobial resistance genes found in airborne dust inside pig farms (*n* = 7) by metagenomic sequencing. (**A)** Proportions of the number of detected genes for each antibiotic class. Proportion represents the percentage of number of detected genes for each antibiotic class to the total number of detected genes of resistance genes of different antibiotic classes from airborne dust collected inside the 7 pig farms. (**B)** Resistance gene profile in airborne dust inside the 7 pig farms. The stacked bar chart represents the number of detected resistance genes in each antibiotic class in each pig farm. Each colored segment within the bars corresponds to a specific resistance gene.

A hundred-fifty nine distinct AMR genes of 12 different antibiotic classes were identified in airborne dust from all pig farms (Table 5), including genes encoding resistance to aminoglycosides (24%), tetracycline (17%), macrolides (16%), β-lactams (13%), folate pathway antagonist (9%), lincomycin (7%), amphenicol (8%), as well as quinolone, polymyxin, glycoprotein, fosfomycin and fusidic acid at 1–2% of each (Figure 3).

**Table 5:** Antimicrobial resistance genes identified in airborne dust inside pig farms (*n* = 159).

**Antimicrobial class**	**Antimicrobial genes**
Aminoglycoside	*aac(3)-IIa, aac(3)-IId, aac(3)-IV, aac(3)-Iva, aac(3)-XI, aac(6')-IIa, aac(6')-Iid, aac(6')-Ib-cr, aac(6')-Ib3, aac(6')-aph(2“), aadA1, aadA11, aadA13, aadA15, aadA17, aadA2, aadA24, aadA2b, aadA3, aadA6, aadA7, aadA8b, aadA9, aadD, ant(2”)-Ia, ant(3“)-Ia, ant(6)-Ia, ant(9)-Ia, aph(2”)-Ia, aph(2“)-Ic, aph(2”)-If, aph(3“)-Ib, aph(3')-III, aph(3')-Ia, aph(3')-Via, aph(6)-Id*
Amphenicol	*cat, cat(pC221), cat86, catA2, catA3, catB2, cfr, cmlA1, cmx, fexA, fexB, floR, optrA*
Beta lactam	*bla_*BRO*−2_*, *bla*_CARB − 2_, *bla*_CARB − 4_, *bla*_CTX − M−14_, *bla*_CTX − M−55_, *bla*_DHA − 1_, *bla*_DHA − 27_, *bla*_EBR − 1_, *bla*_OXA − 10_, *bla*_OXA − 164_, *bla*_OXA − 17_, *bla*_OXA − 209_, *bla*_OXA − 276_, *bla*_OXA − 284_, *bla*_OXA − 347_, *bla*_OXA − 360_, *bla*_OXA − 58_, *bla*_ROB − 1_, *mecA, mecA1, mecB, mecD*,
Folate pathway antagonists	*dfrA1, dfrA12, dfrA14, dfrA15, dfrA16, dfrE, dfrG, sul1, sul2, sul3*
Quinolone	*qnrS1, qnrS10, qnrS11, qnrS13, qnrS3, qnrS8, qnrS9, qnrVC4*
Macrolide	*ere*(D), *erm*(36), *erm*(42), *erm*(45), *erm*(47), *erm*(50), *erm*(A), *erm* (B), *erm*(C), *erm*(F), *erm*(T), *erm*(X), *erm*(Y), *mef*(A), *mef*(C), *mph*(B), *mph*(C), *mph*(E), *mph*(F), *mph*(G), *msr*(A), *msr*(D), *msr*(E)
Tetracyclines	*poxt*A, *tet*(33), *tet*(36), *tet*(39), *tet*(A), *tet*(G), *tet*(H), *tet*(K), *tet*(L), *tet*(M), *tet*(O), *tet*(O/W/32/O), *tet*(S), *tet*(W), *tet*(X), *tet*(X3), *tet*(X4), *tet*(X5), *tet*(X6), *tet*(Y), *tet*(Z), *tetA*(P)
Lincosamide	*Inu*(B)*, Isa*(B)*, Isa*(E)*, sal*(A)*, vga*(A)LC*, vga*(A)V*, vga*(E)
Polymixin	*mcr-1.1, mcr-2.2, mcr3.19, mcr-3.5, mcr-4.3, mcr-6*.
Fusidic acid	*fusC*
Fosfomycin	*fosB, fosB1, fosB4, fosD*
Glycopoptide	*bleO*

Seventy-four resistance genes were identified in ESKAPE species, of which *Staphylococcus* species (32 resistance genes) and *Acinetobacter* species (25 resistance genes) carried most AMR genes. The *mcr* 6.1 gene was detected in *Klebsiella* species, while *Pseudomonas* and *Enterobacter* species did not host any resistance genes as indicated by Bugseq metagenomic sequencing analysis data (Supplementary Table S3).

Several genes encoding resistance to clinically important antibiotics were determined, for example, ß-lactam resistance (e.g., *bla*_CARB − 2_, *bla*_CARB − 4_, *bla*_OXA − 10_, *bla*_OXA − 164_, *bla*_OXA − 17_, *bla*_OXA − 209_, *bla*_OXA − 276_, *bla*_OXA − 284_, *bla*_OXA − 347_, *bla*_OXA − 360_, *bla*_OXA − 58_) and colistin resistance (e.g., *mcr-1.1, mcr-2.2, mcr-3.19, mcr-3.5, mcr-4.3*, and *mcr-6*) (Table 5).

#### 3.4.3 Plasmid diversity

Two hundred-fifty-one plasmids were identified in all pig farms (Supplementary Table S4). Different numbers of plasmids were detected in different farms. The highest number of plasmids were detected in Farm 6 (*n* = 72), followed by Farm 3 (*n* = 56) and Farm 4 (*n* = 53). *S. aureus* (*n* = 48), *A. baumannii* (*n* = 19), and *E. coli* (*n* = 22) served as the most common hosts for the plasmids across all pig farms (Supplementary Table S4). No plasmids were identified in *Pseudomonas* and *Enterobacter*.

The same plasmid was detected in multiple farms, for example, pKKS49 (Accession no. NC_019149), and an unnamed3 plasmid (Accession no. CP027181) were found in Farms 3, 4, 6, 7 and 8. The predicted host of these two plasmids were *S. aureus* and *A. baumannii*, respectively. Another common plasmid is pSALNBL118 (Accession no. CP042023) with the predicted host of *S. aureus* that was found in Farms 3, 4, 5, 6 and 9. Several plasmids carried genes encoding resistance to clinically important antibiotics. For example, *mcr-*carrying plasmids were exclusively found in Farm 6 (*n* = 6), including pCHL5009T-102k-*mcr3* (Accession no. CP032937), pEH_*mcr4.3* (Accession no. CP038261), pMCR3_025943 (Accession no. CP027203), pSa4-CIP (Accession no. MG874042), pTR1 (Accession no. KJ187751), and pYY76–1–2 (Accession no. CP040929) (Supplementary Table S4). These plasmids were hosted by *A. baumannii, E. coli, Salmonella* spp., and *Klebsiella pneumoniae*.

## 4 Discussion

One of the major findings in this study was the presence of various bacterial pathogens and plasmids in airborne dust within and around pig farms. The concentrations of culturable airborne bacteria in pig houses either average (4.8 10^3^ CFU/m^3^) or individual farms (1.9–11.2 × 10^3^ CFU/m^3^) in this study were comparable to a study conducted in farrowing, weaning, and fattening pig houses in China (38) but lower than previous studies in a nursery pig house in South Korea (1.34 × 10^5^ CFU/m3) (39) and in pig confinement facility (1.8 × 10^4^ CFU/m^3^) in the US (22). Thailand has a warm-humid tropical climate throughout the year and the sample collection was taken place in the rainy due to the expectation of high bacterial load inside the farm (23). The quantities of airborne microorganisms are affected by several factors e.g., different animal species, housing arrangements, management practices, and seasonal variations (40). However, the influence of these meteorological parameters was not pursued. The participating pig farms in this study implemented several farm management practices, including close house system with mechanical ventilation, routine cleaning and hygienic maintenance, biosecurity measures, age group separation in different houses, regular health monitoring and disease management systems, all of which helped to lower airborne bacterial counts. The Canadian Occupational Health and Safety Research Institute Robert Sauvé (IRSST) recommended that the concentration of total bacteria present during agricultural activity should not exceed 10,000 CFU/m3 in the air throughout an 8-h work period (41). The bacterial concentrations in all participating pig farms, except Farm 4 (11.2 × 10^3^ CFU/m^3^), were lower than the suggested level. Farm 4 had the most total pigs (13,000 heads), as well as the most pigs (700 heads) in the sampling pen. Typically, larger pig populations in a confined space can result in higher microbial shedding, increased fecal matter and greater respiratory secretions, leading to an increased bacterial load in the air. These factors could explain the observation of the higher bacterial counts in airborne dust in Farm 4 that are greater than the advised level and those in other farms.

*Staphylococcus* has a remarkable ability to adapt their metabolic processes and composition to overcome obstacles, resulting in survival and persistence in an environment where many factors are unfavorable for growth and proliferation (42). Using either a culture-based approach or metagenomic sequencing analysis, *Staphylococcus* species were most prevalent in the airborne dust inside pig farms (Figure 1), in agreement with previous studies (43, 44). In contrast, *Salmonella* species was isolated only from fecal and feed samples, in agreement with previous studies conducted in pig buildings in the US (45) and Eastern Canada (46). The absence of *Salmonella* in airborne dust samples observed was possibly due to the *Salmonella* level being so low that they were undetectable or the culture media being inadequate for culturing airborne samples (46). The latter emphasizes the need for the development of more efficient methods to recover cultivable airborne *Salmonella* species.

It was previously demonstrated that Gram-positive bacteria predominated in airborne microbial contents whereas Gram-negative bacteria were in relatively low numbers (47). This is consistent with the current findings, which found that the most common species in all samples were *Staphylococcus* and *Enterococcus* species. This is likely because Gram-positive can produce spore that enhance their resistance to environmental stresses, leading to better growing in cultivation conditions (48). At the same time, Gram-negative bacteria tend to have short survival times in an airborne environment (49).

Pathogenic bacteria isolated from airborne dust in this study exhibited high AMR rates and most were resistant to multiple drugs (multidrug resistance, MDR), in agreement with previous studies (43, 50). The presence of MDR bacteria in the airborne dust raises a particular concern of the risk of occupational infections, and vice versa, there is a possibility that farm workers serve as asymptomatic reservoirs of MDR bacteria (50, 51). Airborne MDR bacteria have broader public health implications, as they can spread to the community and environment, including people who live close to pig farms.

Despite the limited number, the *Acinetobacter* and *Pseudomonas* isolates displayed resistance to various antimicrobials, e.g., aztreonam, cefotaxime, and ciprofloxacin. These two bacteria are opportunistic pathogens that may inflict diseases in people with impaired lung condition. Clindamycin is commonly used for the treatment of *Staphylococcal* and *Streptococcal* pneumonia, including community-acquired methicillin-resistant *S. aureus* (MRSA) infections (52, 53). Therefore, the presence of clindamycin-resistant *Staphylococcus* species in the airborne dust is of particular concern for public health. Taken together, the findings raise the alarm for the occupational and public health threat associated with airborne dust from pig farms and call for the development of effective measures to minimize the spread of AMR in farm environments.

Metagenomics provides a massive amount of data, yet a lot of it may be irrelevant or ambiguous. In this study, the combination of culture-dependent and -independent techniques was used. The total DNA for metagenomic sequencing analysis was prepared from the pooled bacteria, providing microbiome samples that mostly comprised bacteria DNA rather than other microorganisms. This combination was previously shown to improve understanding of human-associated microbial communities in relation to human health and disease (54). TSA, a non-selective media, contains nutrients required to support the growth of a wide range of bacteria and has been used for culturing bacteria from dust in several previous studies (22, 43, 55). The limitation was that TSA examined only culturable bacteria, resulting in the lack of non-culturable bacteria, fastidious bacteria with high nutrient requirements and bacteria with slow growth rate. The incubation conditions were aerobic; therefore, anaerobic bacteria are overlooked.

Metagenomic sequencing analysis in this study focused on the pooled bacteria collected inside each pig house to investigate the indoor microbial composition and its possible association with antimicrobial usage. Analyzing the microbiome composition of the outdoor environment of pig houses could provide insights into variations in microbial communities but the contributions of variables from other sources must be considered.

Bacillota was a major phylum in this study providing the evidence for the predominance of Gram-positive bacteria in airborne dust. This is in agreement with previous studies demonstrating the presence of the Bacillota in various atmospheric environments including animal farms (56) and animal feces served as a significant source of bacteria in this phylum (57). Therefore, the prolonged storage of manure in pits before its removal could be attributed to the higher bacteria level in the air within pig farms.

Indoor bioaerosols in food animal farms played a crucial role in the dissemination of AMR genes (12), in agreement with the observation of several genes encoding resistance to a wide range of antibiotic classes in this study (Supplementary Table S2). Aminoglycosides resistance encoding genes were at the highest abundance, in agreement with previous studies conducted in urban environment and pig farm (8, 58). A previous study showed that aminoglycoside resistance genes, such as *aph(3*′*)-I, aadE*, and *aad* were dominant in total suspended particulate samples collected from pig and chicken farms (58). The predominance of *aadD, aadE, and aad(6)* was previously observed in bioaerosols in chicken farms (59). High concentrations of *aac6*′*-II, aadA1, aphA3*, and *strB* in the air of various animal farms, e.g., pig, cattle, layer, and broiler farms were recently reported (13). These studies collectively underscore the importance of airborne dust from pig farms as a significant transmission pathway for AMR genes. Interestingly, no resistance genes were detected by Bugseq Metagenomic Sequencing Analysis in *Pseudomonas* and *Enterobacter* species, which are notorious for being MDR and containing multiple AMR genes (Supplementary Table S3). This may be attributed to low DNA content of these two species in airborne samples and the lack of certain AMR genes in the database. Further studies are warranted to explore the mechanisms and implications of AMR transmission through the atmosphere in livestock settings.

Previous studies showed that the global atmosphere is being polluted by AMR genes, in particular β-lactam resistance genes (7) and the occurrence of the genes in airborne particulate matter, dust, and human airways was positively correlated with *Staphylococcus* spp. (60). These agree with the metagenomic sequencing results in this study. A particular concern was the existence of *bla*_OXA_ genes encoding OXA-type carbapenemases known to play a crucial role in carbapenem resistance in *Acinetobacter* and *Pseudomonas* species (61). The airborne transmission of these genes possibly provides the context for the clinical significance and potential treatment challenges in the clinical setting.

Colistin is one of the highest priorities critically important antibiotics but has been extensively used in pig farming. Colistin-resistant *E. coli* strains carrying plasmid-based *mcr-1* were reported in food animals within China in 2016 (62). Since then, various *mcr* variants have been uncovered in livestock populations worldwide. The dissemination of colistin resistance gene including *mcr-1, mcr-2* and *mcr-3* genes were previously reported in Thai pig farms, of which *mcr-3* was most common (63, 64). The Metagenomic Sequence analysis in this study revealed several plasmid-borne colistin resistance genes (e.g., *mcr-1.1, mcr-2.2, mcr-3.19, mcr-3.5, mcr-4.3*, and *mcr-6*). To our knowledge, *mcr-4.3* and *mcr-6* have never been reported in pig farms. No participating farms in this study explicitly mentioned the use of colistin. It is worth noting that colistin was frequently incorporated into medicated feed for suckling and nursery piglets in Thailand as a preventive measure against gastrointestinal tract infections. In Thai pig production, an estimated 40 tons of colistin were combined with medicated feed, of which 87.2% was designated for piglets (65). Extensive use of colistin may lead to the high colistin resistance in the pig production and can spread to the other farms via environmental contamination. The results underline the importance of further studies to comprehend the dynamics and implications of colistin resistance in the interconnected ecosystems of human, animal, and environmental health and to guide the development of effective strategies to preserve the efficacy of this critically important antibiotic.

Based on the metagenomic sequencing analysis, the same plasmids were detected in multiple farms, suggesting the spread of these plasmids among farms. It is well perceived that horizontal transfer of R plasmids plays a crucial role in the wide distribution of AMR. However, *in vitro* horizontal transmission of R plasmids was not pursued in this study.

The plasmids pSALNBL118, pKKS49, and an unnamed plasmid (Accession no. CP027181) were detected in up to 5 farms. pSALNBL118 is a phage like plasmid originated from *S. aureus* strain B3–4A isolated from beef liver (66) and might be important for horizontal gene transfer (67) and transmission of virulence factors. The plasmid pKKS49 carrying *apmA* encoding apramycin resistance, which were originated from an MRSA ST398 isolate obtained from a dust sample taken in a holding with breeding pigs in Portugal (68). Apramycin is frequently used for treatment and control of gastrointestinal tract infection in piglets in Thai pig farms (65), which can create selective pressure leading to resistant of this antibiotic (69). However, it was not disclosed if the antibiotic was used in the participating farm in this study. In addition, the unnamed plasmid carrying *ant (2”)-Ia* was commonly detected in gentamicin-resistant *A. baumannii* strain and should be further characterized due to the clinical importance of the pathogen.

The finding of *mcr*-harboring plasmids was limited to Farm 6, despite the farm not disclosing its use of the antibiotic. This suggests the possibility of contamination from external sources such as farm workers, contaminated feed, and water as well as the environmental contamination of AMR bacteria and genes, in addition to co-selection by other antibiotics. pCHL5009T-102k-*mcr3* harboring *mcr-3.5* and *bla*_CTX − M55_ was identified in *E. coli*. The plasmid was originally found to carry both *mcr-1* and *mcr-3* and isolated from *E. coli* in a patient in New Zealand who have experienced travel to Thailand (70). The observation of pCHL5009T-102k-*mcr3* corresponded to previous studies that revealed the limited presence of *mcr-1* and the predominance of *mcr-3* in the *E. coli* isolates from healthy and sick pigs (64). Even though the reason underlying for the *mcr-1* loss remains unclear, the co-localization of *mcr* and *bla*_CTX − M55_ on the same plasmid is an alarm for the distribution of bacterial pathogens resistant to last line antibiotics in the airborne dust in pig farm (71).

Additional limitations to this study are noted. Despite significant advancements in genomics, many resistance genes and bacterial species are still unidentified and unrepresented in databases. Furthermore, some bacteria with low biomass and resistance genes with low copies may not yield sufficient DNA copies for identifying by Metagenomic sequencing.

In conclusion, the results in this study demonstrated that the airborne dust around and within pig houses contained a wide array of microorganisms as well as AMR bacteria, posing potential risks to human, animal, and environmental health. The use of culture-based techniques in combination with metagenomic sequencing analysis enables the detection of a wider range of bacteria and their AMR genotype, providing valuable insights and providing crucial information for risk assessment, intervention planning, and informed decision-making to combat the spread of AMR. Specific guidelines to limit bioaerosol concentrations in livestock farm as well as to protect worker health are required. Future studies are suggested to examine the impact of AMR-contaminated airborne dust exposures in areas where animals are reared on the general public's health.

## Data availability statement

Metagenomic sequencing data were submitted to NCBI Sequence Read Archive (SRA) with accession numbers SRR25743046–SRR25743052 under the BioProject accession number PRJNA1006161.

## Author contributions

SH: Conceptualization, Formal analysis, Investigation, Methodology, Visualization, Writing—original draft, Writing—review & editing. RP: Investigation, Writing—review & editing. SS: Investigation, Writing—review & editing. DM: Investigation, Methodology, Writing—review & editing. TW: Formal analysis, Software, Visualization, Writing—review & editing. PJ: Formal analysis, Software, Visualization, Writing—review & editing. PT: Investigation, Methodology, Writing—review & editing. RC: Conceptualization, Data curation, Formal analysis, Funding acquisition, Investigation, Methodology, Project administration, Resources, Supervision, Validation, Visualization, Writing—original draft, Writing—review & editing.

## References

[1] UNEP. Frontiers 2017 Emerging Issues of Environmental Concern. United Nations Environment Programme Nairobi (2017).

[2] Wang J Qin X Guo J Jia W Wang Q Zhang M. Evidence of selective enrichment of bacterial assemblages and antibiotic resistant genes by microplastics in urban rivers. Water Res. (2020) 183:116113. 10.1016/j.watres.2020.116113 32668354

[3] D'Costa VM King CE Kalan L Morar M Sung WW Schwarz C Froese D. Antibiotic resistance is ancient. Nature. (2011) 477:457–61. 10.1038/nature10388 21881561

[4] Chen Q. Long-term field application of sewage sludge increases the abundance of antibiotic resistance genes in soil. Environ Int. (2016) 92:1–10. 10.1016/j.envint.2016.03.026 27043971

[5] Yang C Zhang W Liu R Li Q Li B Wang S. Phylogenetic diversity and metabolic potential of activated sludge microbial communities in full-scale wastewater treatment plants. Environ Sci Technol. (2011) 45:7408–15. 10.1021/es2010545 21780771

[6] Rodriguez-Mozaz S Chamorro S Marti E Huerta B Gros M Sànchez-Melsió A. Occurrence of antibiotics and antibiotic resistance genes in hospital and urban wastewaters and their impact on the receiving river. Water Res. (2015) 69:234–42. 10.1016/j.watres.2014.11.021 25482914

[7] Li J Cao J Zhu Y-g Chen Q-l Shen F Wu Y. Global survey of antibiotic resistance genes in air. Environ Sci Technol. (2018) 52:10975–84. 10.1021/acs.est.8b02204 30043612

[8] Zhu G Wang X Yang T Su J Qin Y Wang S. Air pollution could drive global dissemination of antibiotic resistance genes. ISME J. (2021) 15:270–81. 10.1038/s41396-020-00780-2 32963346 PMC7852678

[9] Gao M Zhang X Yue Y Qiu T Wang J Wang X. Air path of antimicrobial resistance related genes from layer farms: emission inventory, atmospheric transport, and human exposure. J Hazard Mater. (2022) 430:128417. 10.1016/j.jhazmat.2022.128417 35183825

[10] Denissen J Reyneke B Waso-Reyneke M Havenga B Barnard T Khan S. Prevalence of eskape pathogens in the environment: antibiotic resistance status, community-acquired infection and risk to human health. J Hyg Environ. (2022) 244:114006. 10.1016/j.ijheh.2022.114006 35841823

[11] Muntean MM Muntean A-A Preda M Manolescu LSC Dragomirescu C Popa M-I. Phenotypic and genotypic detection methods for antimicrobial resistance in eskape pathogens. Exp Ther Med. (2022) 24:1–12. 10.3892/etm.2022.11435 35837033 PMC9257796

[12] Bai H He L-Y Wu D-L Gao F-Z Zhang M Zou H-Y. Spread of airborne antibiotic resistance from animal farms to the environment: dispersal pattern and exposure risk. Environ Int. (2022) 158:106927. 10.1016/j.envint.2021.106927 34673316

[13] Xin H Gao M Wang X Qiu T Guo Y Zhang L. Animal farms are hot spots for airborne antimicrobial resistance. Sci Total Environ. (2022) 851:158050. 10.1016/j.scitotenv.2022.158050 35985594

[14] Song L Wang C Jiang G Ma J Li Y Chen H. Bioaerosol is an important transmission route of antibiotic resistance genes in pig farms. Environ Int. (2021) 154:106559. 10.1016/j.envint.2021.106559 33864959

[15] McEachran AD Blackwell BR Hanson JD Wooten KJ Mayer GD Cox SB. Antibiotics, bacteria, and antibiotic resistance genes: aerial transport from cattle feed yards via particulate matter. Environ Health Perspect. (2015) 123:337–43. 10.1289/ehp.1408555 25633846 PMC4383574

[16] Casey JA Kim BF Larsen J Price LB Nachman KE. Industrial food animal production and community health. Curr Environ Health Rep. (2015) 2:259–71. 10.1007/s40572-015-0061-0 26231503 PMC13570458

[17] Munk P Knudsen BE Lukjancenko O Duarte ASR Van Gompel L Luiken RE. Abundance and diversity of the faecal resistome in slaughter pigs and broilers in nine european countries. Nat Microbiol. (2018) 3:898–908. 10.1038/s41564-018-0192-9 30038308

[18] Tiseo K Huber L Gilbert M Robinson TP Van Boeckel TP. Global trends in antimicrobial use in food animals from 2017 to 2030. Antibiotics. (2020) 9:918. 10.3390/antibiotics9120918 33348801 PMC7766021

[19] Lekagul A Kirivan S Tansakul N Krisanaphan C Srinha J Laoprasert T. Antimicrobial consumption in food-producing animals in thailand between 2017 and 2019: the analysis of national importation and production data. PLoS ONE. (2023) 18:e0283819. 10.1371/journal.pone.0283819 37104254 PMC10138855

[20] Luiken RE Heederik DJ Scherpenisse P Van Gompel L van Heijnsbergen E Greve GD. Determinants for antimicrobial resistance genes in farm dust on 333 poultry and pig farms in nine European countries. Environ Res. (2022) 208:112715. 10.1016/j.envres.2022.112715 35033551

[21] Chaivisit P Fontana A Galindo S Strub C Choosong T Kantachote D. Airborne bacteria and fungi distribution characteristics in natural ventilation system of a university hospital in Thailand. Environ Asia. (2018) 11:2. 10.14456/ea.2018.22

[22] Green CF Gibbs SG Tarwater PM Mota LC Scarpino PV. Bacterial plume emanating from the air surrounding swine confinement operations. J Occup Environ Hyg. (2006) 3:9–15. 10.1080/15459620500430615 16482973

[23] Kumar P Singh A Singh R. Seasonal variation and size distribution in the airborne indoor microbial concentration of residential houses in Delhi and its impact on health. Aerobiologia. (2021) 37:719–32. 10.1007/s10453-021-09718-3 34248257 PMC8254435

[24] EFSA. Report from the task force on zoonoses data collection including guidance for harmonized monitoring and reporting of antimicrobial resistance in commensal *Escherichia Coli* and *Enterococcus* Spp. from food animals. EFSA J. (2008) 6:141r. 10.2903/j.efsa.2008.141r

[25] Ke D Picard FoJ Martineau F Ménard C Roy PH Ouellette M Bergeron MG. Development of a PCR assay for rapid detection of *Enterococci*. J Clin Microbiol. (1999) 37:3497–503. 10.1128/JCM.37.11.3497-3503.1999 10523541 PMC85677

[26] Sharp SE Searcy C. Comparison of mannitol salt agar and blood agar plates for identification and susceptibility testing of *Staphylococcus Aureus* in specimens from cystic fibrosis patients. J Clin Microbiol. (2006) 44:4545–6. 10.1128/JCM.01129-06 17021065 PMC1698434

[27] Dziri R Klibi N Alonso CA Said LB Bellaaj R Slama KB. Characterization of extended-spectrum B-lactamase (Esbl)-producing *Klebsiella, Enterobacter*, and *Citrobacter* obtained in environmental samples of a Tunisian hospital. Diagn Microbiol Infect Dis. (2016) 86:190–3. 10.1016/j.diagmicrobio.2016.07.013 27492133

[28] Younis A Elbialy A Abo Remila E Ammar A. Molecular detection of genus *Klebsiella* and genotypic identification of *Klebsiella Pneumoniae* and *Klebsiella Oxytoca* by duplex polymerase chain reaction in poultry. Glob Vet. (2017) 18:234–41. 10.5829/idosi.gv.2017.234.241

[29] Anane A Y Apalata T Vasaikar S Okuthe GE Songca S. Prevalence and molecular analysis of multidrug-resistant *Acinetobacter baumannii* in the extra-hospital environment in Mthatha, South Africa. Braz J Infect Dis. (2020) 23:371–80. 10.1016/j.bjid.2019.09.004 31706742 PMC9428220

[30] Ghane M Azimi Z. Isolation, Identification and antimicrobial susceptibility of *Pseudomonas* Spp. isolated from hospital environment in Tonekabon, North of Iran. Appl Environ Microbiol. (2014) 2:97–101. 10.12691/jaem-2-4-2

[31] Asselin JAE Bonasera JM Beer SV. Pcr primers for detection of pantoea ananatis, *Burkholderia* Spp, and *Enterobacter* Sp from onion. Plant Dis. (2016) 100:836–46. 10.1094/PDIS-08-15-0941-RE 30688614

[32] Feng P Weagant SD Grant MA Burkhardt W Shellfish M Water B. Bam: Enumeration of Escherichia Coli and the Coliform Bacteria. (2002), 1–13.

[33] ISO. Microbiology of Food and Animal Feeding Stuffs. Horizontal Method for the Detection of Salmonella Spp. Amendment 1: Anex D: Detection of Salmonella Spp. In Animal Faeces and in Environmental Samples from the Primary Production Stage. Geneva: International Standard Organization (2007).

[34] Meiri-Bendek I Lipkin E Friedmann A Leitner G Saran A Friedman S. PCR-based method for the detectionof *Streptococcus Agalactiae* in milk. J Dairy Sci. (2002) 85:1717–23. 10.3168/jds.S0022-0302(02)74245-8 12201522

[35] Wyder AB Boss R Naskova J Kaufmann T Steiner A Graber HU. *Streptococcus* Spp. and related bacteria: their identification and their pathogenic potential for chronic mastitis–a molecular approach. Res Vet Sci. (2011) 91:349–57. 10.1016/j.rvsc.2010.09.006 20971488

[36] CLSI. Performance Standards for Antimicrobial Disk and Dilution Susceptibility Tests for Bacteria Isolated from Animals. Wayne, PA: Clinical and Laboratory Standards Institute (2018).

[37] Fan J Huang S Chorlton SD. Bugseq: a highly accurate cloud platform for long-read metagenomic analyses. BMC Bioinform. (2021) 22:1–12. 10.1186/s12859-021-04089-5 33765910 PMC7993542

[38] Cui H Zhang C Liu J Dong S Zhao K Chen L. The distribution characteristics of aerosol bacteria in different types of pig houses. Animals. (2022) 12:1540. 10.3390/ani12121540 35739876 PMC9219456

[39] Jo W-K Kang J-H. Exposure levels of airborne bacteria and fungi in korean swine and poultry sheds. Arch Environ Occup Health. (2005) 60:140–6. 10.3200/AEOH.60.3.140-146 17153086

[40] Zhao Y Aarnink AJ De Jong MC Groot Koerkamp PW. Airborne microorganisms from livestock production systems and their relation to dust. Crit Rev Environ Sci Technol. (2014) 44:1071–128. 10.1080/10643389.2012.746064 32288664 PMC7113898

[41] Goyer N Lavoie J Lazure L Marchand G. Bioaerosols in the Workplace: Evaluation, Control and Prevention Guide. London: Institut de recherche Robert-Sauvé en santé et en sécurité du travail (2001).

[42] Onyango LA Alreshidi MM. Adaptive metabolism in staphylococci: survival and persistence in environmental and clinical settings. J Patho. (2018) 2018:2632. 10.1155/2018/1092632 30327733 PMC6171259

[43] Gibbs SG Green CF Tarwater PM Mota LC Mena KD Scarpino PV. Isolation of antibiotic-resistant bacteria from the air plume downwind of a swine confined or concentrated animal feeding operation. Environ Health Perspect. (2006) 114:1032–7. 10.1289/ehp.8910 16835055 PMC1513331

[44] Predicala BZ Urban JE Maghirang RG Jerez SB Goodband RD. Assessment of bioaerosols in swine barns by filtration and impaction. Curr Microbiol. (2002) 44:136–40. 10.1007/s00284-001-0064-y 11815859

[45] Elliott L McCalla T Deshazer J. Bacteria in the air of housed swine units. Appl Environ Microbiol. (1976) 32:270–3. 10.1128/aem.32.2.270-273.1976 16345169 PMC170047

[46] Pilote J Létourneau V Girard M Duchaine C. Quantification of airborne dust, endotoxins, human pathogens and antibiotic and metal resistance genes in eastern canadian swine confinement buildings. Aerobiologia. (2019) 35:283–96. 10.1007/s10453-019-09562-6

[47] Cormier Y Tremblay G Meriaux A Brochu G Lavoie J. Airborne microbial contents in two types of swine confinement buildings in Quebec. Am Ind Hyg Assoc. (1990) 51:304–9. 10.1202/0002-8894(1990)051&lt;0304:AMCITT&gt;2.0.CO;2 2353639

[48] Ruiz-Gil T Acuña JJ Fujiyoshi S Tanaka D Noda J Maruyama F. Airborne bacterial communities of outdoor environments and their associated influencing factors. Environ Inter. (2020) 145:106156. 10.1016/j.envint.2020.106156 33039877

[49] Zucker BA Trojan S Müller W. Airborne gram-negative bacterial flora in animal houses. J Vet Med. (2000) 47:37–46. 10.1046/j.1439-0450.2000.00308.x 10780171

[50] Chapin A Rule A Gibson K Buckley T Schwab K. Airborne multidrug-resistant bacteria isolated from a concentrated swine feeding operation. Environ Health Perspect. (2005) 113:137–42. 10.1289/ehp.7473 15687049 PMC1277855

[51] Just NA Létourneau V Kirychuk SP Singh B Duchaine C. Potentially pathogenic bacteria and antimicrobial resistance in bioaerosols from cage-housed and floor-housed poultry operations. Ann Occup Hyg. (2012) 56:440–9. 10.1093/annhyg/mer105 22156572

[52] Herigon JC Hersh AL Gerber JS Zaoutis TE Newland JG. Antibiotic management of *Staphylococcus Aureus* infections in US children's hospitals, 1999–2008. Pediatrics. (2010) 125:e1294–e300. 10.1542/peds.2009-2867 20478934

[53] Marcinak JF Frank AL. Treatment of community-acquired methicillin-resistant *Staphylococcus Aureus* in children. Curr Opin Infect Dis. (2003) 16:265–9. 10.1097/00001432-200306000-00014 12821819

[54] Whelan FJ Waddell B Syed SA Shekarriz S Rabin HR Parkins MD. Culture-enriched metagenomic sequencing enables in-depth profiling of the cystic fibrosis lung microbiota. Nat Microbio. (2020) 5:379–90. 10.1038/s41564-019-0643-y 31959969

[55] Vantarakis A Paparrodopoulos S Kokkinos P Vantarakis G Fragou K Detorakis I. Impact on the quality of life when living close to a municipal wastewater treatment plant. J Environ Public Health. (2016) 2016:7023. 10.1155/2016/8467023 27375747 PMC4914737

[56] de Groot GA Geisen S Wubs EJ Meulenbroek L Laros I Snoek LB. The aerobiome uncovered: multi-marker metabarcoding reveals potential drivers of turn-over in the full microbial community in the air. Environ Int. (2021) 154:106551. 10.1016/j.envint.2021.106551 33857708

[57] Gao X-L Shao M-F Wang Q Wang L-T Fang W-Y Ouyang F. Airborne microbial communities in the atmospheric environment of urban hospitals in China. J Hazard Mater. (2018) 349:10–7. 10.1016/j.jhazmat.2018.01.043 29414740

[58] Yang Y Zhou R Chen B Zhang T Hu L Zou S. Characterization of airborne antibiotic resistance genes from typical bioaerosol emission sources in the urban environment using metagenomic approach. Chemosphere. (2018) 213:463–71. 10.1016/j.chemosphere.2018.09.066 30245223

[59] Yang F Gao Y Zhao H Li J Cheng X Meng L. Revealing the distribution characteristics of antibiotic resistance genes and bacterial communities in animal-aerosol-human in a chicken farm: from one-health perspective. Ecotoxicol Environ Saf. (2021) 224:112687. 10.1016/j.ecoenv.2021.112687 34438267

[60] Zhou Z-C Liu Y Lin Z-J Shuai X-Y Zhu L Xu L. Spread of antibiotic resistance genes and microbiota in airborne particulate matter, dust, and human airways in the urban hospital. Environ Int. (2021) 153:106501. 10.1016/j.envint.2021.106501 33836339

[61] Evans BA Amyes SG. Oxa B-Lactamases. Clin Microbiol Rev. (2014) 27:241–63. 10.1128/CMR.00117-13 24696435 PMC3993105

[62] Liu Y-Y Wang Y Walsh TR Yi L-X Zhang R Spencer J. Emergence of plasmid-mediated colistin resistance mechanism Mcr-1 in animals and human beings in China: a microbiological and molecular biological study. Lancet Infect Dis. (2016) 16:161–8. 10.1016/S1473-3099(15)00424-7 26603172

[63] Lay KK Jeamsripong S Sunn KP Angkititrakul S Prathan R Srisanga S. Colistin resistance and Esbl production in *Salmonella* and *Escherichia Coli* from Pigs and Pork in the Thailand, Cambodia, Lao Pdr, and Myanmar border area. Antibiotics. (2021) 10:657. 10.3390/antibiotics10060657 34072965 PMC8226727

[64] Trongjit S Assavacheep P Samngamnim S My TH An VTT Simjee S. Plasmid-mediated colistin resistance and Esbl production in *Escherichia Coli* from clinically healthy and sick pigs. Sci Rep. (2022) 12:2466. 10.1038/s41598-022-06415-0 35165337 PMC8844364

[65] Lekagul A Tangcharoensathien V Mills A Rushton J Yeung S. How Antibiotics Are Used in Pig Farming: A Mixed-Methods Study of Pig Farmers, Feed Mills and Veterinarians in Thailand. BMJ Glob Health. (2020) 5:e001918. 10.1136/bmjgh-2019-001918 32180998 PMC7050320

[66] Karki AB Neyaz L Fakhr MK. Comparative genomics of plasmid-bearing *Staphylococcus Aureus* strains isolated from various retail meats. Front Microbiol. (2020) 11:574923. 10.3389/fmicb.2020.574923 33193185 PMC7644949

[67] Goerke C Pantucek R Holtfreter S Schulte B Zink M Grumann D. Diversity of prophages in dominant *Staphylococcus Aureus* clonal lineages. J bacteriol. (2009) 191:3462–8. 10.1128/JB.01804-08 19329640 PMC2681900

[68] Kadlec K Feßler AT Couto N Pomba CF Schwarz S. Unusual small plasmids carrying the novel resistance genes *Dfrk* or *Apma* isolated from methicillin-resistant or-susceptible staphylococci. J Antimicrob Chemother. (2012) 67:2342–5. 10.1093/jac/dks235 22718530

[69] Luppi A. Swine enteric colibacillosis: diagnosis, therapy and antimicrobial resistance. Porcine Health Manag. (2017) 3:1–18. 10.1186/s40813-017-0063-4 28794894 PMC5547460

[70] Creighton J Anderson T Howard J Dyet K Ren X Freeman J. Co-Occurrence of *Mcr-1* and *Mcr-3* genes in a single *Escherichia Coli* in New Zealand. J Antimicrob Chemother. (2019) 74:3113–6. 10.1093/jac/dkz311 31339995

[71] Pungpian C Angkititrakul S Chuanchuen R. Genomic characterization of antimicrobial resistance in *Mcr*-carrying Esbl-producing *Escherichia Coli* from pigs and humans. Microbiology. (2022) 168:001204. 10.1099/mic.0.001204 35766988

